# Visual outcomes following endoscopic endonasal approach for tuberculum sellae meningiomas: a systematic review and meta-analysis

**DOI:** 10.1097/JS9.0000000000004737

**Published:** 2026-01-19

**Authors:** Cheng-Chi Lee, Ting-Wei Chang, Yu-Chi Wang, Ya-Jui Lin, Yin-Cheng Huang, Peng-Wei Hsu, Abel Po-Hao Huang, Chi-Cheng Chuang

**Affiliations:** a Department of Neurosurgery, Chang Gung Memorial Hospital at Linkou and Chang Gung University, Taoyuan, Taiwan; b Department of Biomedical Engineering, National Taiwan University, Taipei, Taiwan; c Program of Biomedical Engineering, Graduate Institute of Biomedical Engineering, Department of Biomedical Engineering, College of Engineering, Chang Gung University, Taoyuan, Taiwan; d Graduate Institute of Clinical Medical Sciences, College of Medicine, Chang Gung University, Taoyuan, Taiwan; e School of Medicine, National Tsing Hua University, Hsinchu, Taiwan; f Division of Neurosurgery, Department of Surgery, National Taiwan University Hospital, National Taiwan University College of Medicine, Taipei, Taiwan; g Institute of Polymer Science and Engineering, National Taiwan University, Taipei, Taiwan

**Keywords:** endoscopic endonasal approach, meta-analysis, systematic review, transcranial approach, tuberculum sellae meningioma, visual outcome

## Abstract

**Background and objectives:**

This systematic review and meta-analysis assessed visual outcomes following tuberculum sellae meningiomas (TSM) resection using the endoscopic endonasal approach (EEA). TSM are frequently managed surgically, with vision restoration or preservation as a primary goal.

**Methods:**

A systematic search of PubMed, EMBASE, and Cochrane CENTRAL identified studies published before September 2024 that reported postoperative vision status following TSM resection via EEA, with or without comparisons to traditional transcranial approach (TCA). Primary outcomes included rates of improved or stable vision and improved vision alone after EEA. Secondary outcomes compared EEA and TCA visual results. Subgroup and sensitivity analyses were performed to explore the source of heterogeneity. Analyses were conducted using R Studio (PROSPERO: [blinded for review]).

**Results:**

Thirty-two retrospective studies were included, covering 1174 TSM patients receiving EEA and 1199 receiving TCA. Preoperative visual impairment was reported in 56%–100% of patients. After EEA, the pooled rate of improved or stabilized vision, including both patients with improvement and those whose vision remained stable, was 0.93 (95% CI: 0.90–0.94). The rate of improved vision alone was 0.77 (95% CI: 0.71–0.82). Compared to TCA, EEA demonstrated significantly higher odds of achieving visual improvement or stability (OR: 2.09, 95% CI: 1.41–3.08) and improved vision alone (OR: 2.28, 95% CI: 1.77–2.93). No significant publication bias was detected.

**Conclusion:**

EEA appears to be an effective approach for achieving favorable visual outcomes in patients with TSM. While pooled data suggest a potential advantage over TCA, these findings should be interpreted cautiously given the limitations of the available retrospective evidence. Further prospective studies with standardized visual assessments are needed to confirm these findings.

## Introduction

Tuberculum sellae meningiomas (TSM) are cranial tumors situated at the midline skull base in close anatomical proximity to the optic chiasm and optic nerves^[1]^. Beyond their association with visual structures, the tuberculum sellae region is flanked by critical arterial supply to the hypothalamus, presenting significant challenges for both radiotherapy and surgical intervention^[2,3]^.

Due to their proximity to the optic chiasm, TSMs frequently compress the optic nerves, resulting in visual disturbances, including visual field deficits and reduced visual acuity. These visual impairments represent the principal morbidity of symptomatic TSMs, with up to 80% of patients experiencing self-reported or clinically assessed visual dysfunction^[2,4–7]^. The primary objective in TSM management is to preserve or restore visual function while achieving maximal tumor resection. Prognostic classifications, such as the Magill-McDermott (M-M) scale, have been developed to guide surgical decision-making and predict postoperative outcomes based on tumor characteristics^[2,8–10]^. The M-M scale incorporates tumor size, optic canal invasion, and arterial encasement and has been validated in multicenter cohorts as a predictor of visual outcomes and the likelihood of gross total resection (GTR)^[2,8]^. Furthermore, preoperative visual acuity and the duration of symptoms have been correlated with postoperative visual recovery^[9,10]^. However, despite guidelines advocating standardized visual assessments, reporting remains inconsistent, often relying on subjective classifications of visual improvement, stabilization, or worsening, with limited use of formalized evaluation metrics^[5,7]^.

Surgical resection remains the cornerstone of TSM management for symptomatic cases^[5]^. Recent advancements, particularly in vascularized nasoseptal flap techniques, have facilitated the adoption of the minimally invasive endoscopic endonasal approach (EEA) as an alternative to traditional transcranial approaches (TCAs).^[5–7]^ EEA provides direct access to the tumor while avoiding brain tissue retraction^[3]^, potentially resulting in fewer complications and shorter hospitalizations. A recent survey indicated a substantial increase in the use of EEA for TSM resections, rising from negligible use in 2006 to approximately 50% of cases by 2019^[7]^. Clinically, EEA is particularly suited for midline TSMs with favorable anatomy, while vessel encasement or optic canal invasion may limit its applicability^[11]^. Studies have increasingly demonstrated superior postoperative visual outcomes with EEA, with GTR rates and recurrence rates comparable to those achieved with TCA^[12–15]^.

Despite increasing research on EEA, substantial variability persists in how visual outcomes are defined and reported. Moreover, existing meta-analyses have often failed to account for the lack of standardized visual outcomes and formal visual assessments – whether subjective or objective – thereby limiting the comparability and reliability of their conclusions^[13,15]^. This necessitates a systematic review and meta-analysis to consolidate the evidence and clarify the ophthalmologic impact of TSM surgical management. In this study, we aim to address this gap through a comprehensive review of published data on postoperative visual outcomes associated with EEA for TSMs. We synthesized data to evaluate the rates of visual improvement or stabilization following EEA and conducted comparisons with TCA where data were available. Through these analyses, we seek to enhance the understanding of EEA’s role in the surgical management of TSMs and to inform future clinical practice and research in this evolving domain. This systematic review complies with the TITAN 2025 (Transparency in the Reporting of Artificial Intelligence) guidelines for responsible artificial intelligence (AI) use and reporting^[16]^.

## Methods

### Search strategy

This systematic review and meta-analysis was registered in PROSPERO (registration number: [CRD420251002845]) and conducted in accordance with the Preferred Reporting Items for Systematic Reviews and Meta-Analyses guidelines^[17]^. A comprehensive search of PubMed, EMBASE, and CENTRAL databases was conducted to identify studies published from inception up to 6 September 2024. The search strategy combined controlled vocabulary terms, that is, Medical Subject Headings, with free-text keywords and Boolean operators to construct the search string: [*(visual OR vision OR optic) AND (endoscopic OR endonasal OR transnasal)] AND [(“tuberculum sellae*”) AND (meningioma)]. This search string was used across the three databases. To ensure comprehensiveness, reference lists of included studies were manually screened to identify additional relevant publications not captured in the electronic search. The methodological quality of this meta-analysis was assessed using the AMSTAR-2 (Assessing the methodological quality of systematic reviews-2) checklist, which evaluates 16 domains related to the planning, conduct, and reporting of systematic reviews^[18]^.

**HIGHLIGHTS**

- **One of the most comprehensive analyses –** Among the few to synthesize visual outcomes in tuberculum sellae meningiomas after endoscopic endonasal approach (EEA).
- **High visual success with EEA** – 93% improved or stable vision; 77% improved.
- **Trend toward better outcomes vs** transcranial approach **–** Higher pooled odds in available studies.
- **Evidence supports EEA as a viable option** – Consistently favorable visual outcomes across multiple studies, though heterogeneity limits certainty.

### Selection criteria

Studies were selected based on the PICOS framework^[19]^. The population of interest consisted of patients (P) diagnosed with TSM who underwent surgical resection. The intervention (I) was EEA, with or without a comparison group treated via TCA. Postoperative visual outcomes were the primary outcomes (O) of interest, while eligible study designs (S) included randomized controlled trials (RCTs) as well as prospective and retrospective cohort studies, either single- or two-armed. Studies were excluded if they were review articles, letters, editorials, conference abstracts, surgical technical notes, case reports, or included fewer than 10 patients. Preclinical studies and those without quantitative visual outcomes were also excluded.

### Main outcome measures

The primary outcomes of this meta-analysis were the pooled rates of (1) improved or stable postoperative vision and (2) improved postoperative vision following TSM resection via EEA. The secondary outcomes were, for studies including a comparison group of TCA, the odds ratios (ORs) for achieving (1) improved or stable postoperative vision and (2) improved vision, when comparing EEA to TCA.

Notably, the included studies used the terms “improved” and “stable” vision with criteria that were often not reported, making it unclear whether their definitions were consistent across studies. We therefore retained the classifications exactly as reported in the original studies. None of the studies provided standardized quantitative thresholds or raw visual data that would allow harmonization across studies. To manage this variability, we prespecified two pooled outcomes – (1) improved or stable vision and (2) improved vision alone – based strictly on the categories defined in each study. In the absence of uniform ophthalmologic metrics, further transformation or standardization of these outcome categories was not feasible.

### Data extraction

Two independent reviewers screened studies for eligibility using the predefined criteria, with a third reviewer consulted in cases of disagreement. From eligible studies, extracted variables included the authors and year of publication, geographic region, total number of patients, group-specific sample sizes (EEA *vs* TCA), and demographic characteristics such as age and sex distribution. Clinical data were also recorded, including the prevalence of preoperative visual impairment, headache, optic canal involvement, mean preoperative tumor volume, GTR rates, and postoperative visual outcomes. Data were tabulated and organized using Microsoft Excel to ensure accuracy and facilitate analysis.

### Quality assessment

The quality of the comparative studies was assessed using the Newcastle-Ottawa Scale (NOS), a tool endorsed by the Cochrane Non-Randomized Studies Methods Working Group for evaluating non-randomized studies in meta-analyses^[20]^. The cohort version of the NOS was used, assessing nine criteria in selection, comparability, and outcome domains. Studies can score up to 9 stars, with 7–9 indicating high quality and 4–6 medium quality. Additionally, the Modified 18-item Delphi checklist was employed to assess the quality of all the single-arm studies included uniformly^[21]^. Two independent reviewers performed the quality assessment, and any disagreement was resolved through discussion.

### Statistical analysis

Pooled event rates with 95% confidence intervals (CIs) were calculated for the primary outcomes, while ORs with 95% CIs were used for secondary outcomes. An OR >1 favored EEA, while an OR <1 favored TCA for visual outcomes. Heterogeneity was assessed using the chi-square test (χ^2^), inconsistency index (*I*^2^), and *Q* statistic. Based on *I*^2^ values, heterogeneity was classified as low (*I*^2^ ≤ 25%), moderate (25% < *I*^2^ ≤ 50%), substantial (50% < *I*^2^ ≤ 75%), or high (*I*^2^ > 75%). Random-effects models were used when moderate to high heterogeneity (*I*^2^ > 50%) was observed, as they account for both within-study and between-study variability, providing more conservative and generalizable estimates across diverse study populations and methodologies. Fixed-effects models were applied only when heterogeneity was minimal or absent, assuming that all included studies estimate the same underlying effect. A two-sided *P*-value of less than 0.05 was considered statistically significant.

Subgroup analyses explored potential sources of heterogeneity, stratifying studies by geographic region (Europe/Americas *vs* Asia) and publication period (before *vs* after 2015). Sensitivity analyses were conducted using the leave-one-out method, whereby each study was sequentially removed to assess its influence on the pooled estimates. Publication bias was evaluated through visual inspection of funnel plot symmetry and Egger’s test^[22]^. The absence of significant bias was inferred from symmetric funnel plots and one-sided *P* > 0.05^[23]^.

All statistical analyses were performed using R Studio version 4.3.2, employing the “meta,” “dmetar,” and “metafor” packages.^[24–26]^

### Compliance with TITAN guidelines

This review adheres to the 2025 TITAN guidelines. Generative AI (ChatGPT, GPT-4; OpenAI, San Francisco, CA, USA) was used only for language editing of the manuscript text (grammar and clarity) under direct human supervision. No AI was used for study design, data analysis, or generation of original scientific content. Date used: February 2025; configuration: standard interface, no plug-ins or fine-tuning, and default temperature; access via cloud-based web service. Only manuscript text was provided, with no patient or confidential data, and all inputs complied with privacy regulations. The corresponding author reviewed all outputs for accuracy and revised or discarded any unsuitable text.

## Results

### Literature search

**Figure 1.**
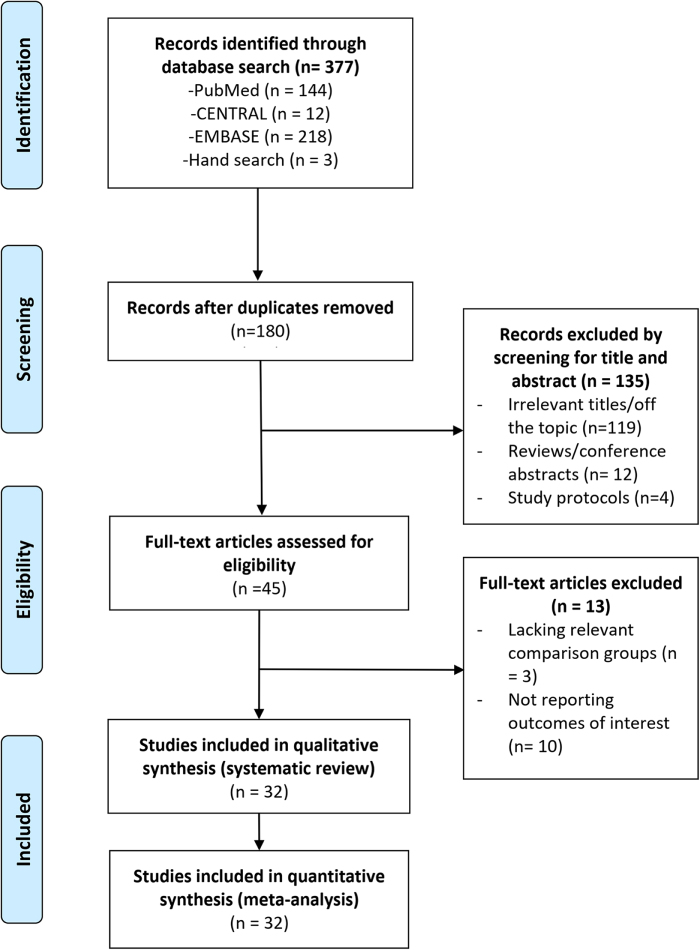
PRISMA flow diagram of study selection process.

Figure 1 depicts the electronic literature search and study selection process. From 377 records identified, 180 non-duplicated studies underwent content screening. Following title and abstract review, 135 studies were excluded for being irrelevant to the topic or inappropriate in publication type. Of the 45 studies subjected to full-text review, 13 were excluded for lacking outcomes of interest or involving irrelevant comparison groups. Consequently, 32 studies satisfied the inclusion criteria and were incorporated into the systematic review and meta-analysis^[7,9,27–56]^.

### Study characteristics and systematic review of visual assessment

Table 1 outlines the characteristics of studies included for meta-analysis. Among these, 19 studies compared EEA to TCA, encompassing 1958 patients (759 treated with EEA and 1199 with TCA). Additionally, 13 single-arm studies focused on EEA outcomes, comprising 415 patients. In total, 1174 patients underwent EEA. All studies were retrospective, with none classified as RCTs. The mean patient age ranged from 41 to 62 years, with the proportion of male patients varying from 3% to 40%. Impaired preoperative vision was reported in 56%–100% of patients, while headache was reported by 1.2%–42.3% of patients. Prior to surgery, optical canal involvement was identified in none to 92.3% of patients, while GTR rates ranged from 53% to 95% postoperatively. Geographically, 17 studies were conducted in Asian countries (e.g., Japan, China, Korea, India, and Bangladesh), while the remainder originated from countries in Europe or the Americas (e.g., Canada, Italy, Mexico, Turkey, and the US). Most studies reported pre- and postoperative ophthalmologic assessments of visual acuity and visual field, typically performed by specialists^[9,31–34,38–41,44,46–48,50–56]^. Visual acuity was commonly assessed using the Snellen chart, while visual field testing employed Goldmann perimetry or Humphrey visual field tests^[9,28,39,40,45,47,51–54]^. The German Ophthalmology Society’s visual impairment score was the most frequently utilized tool to evaluate visual impairment and postoperative changes^[9,40,45,52–54]^. However, inconsistencies were noted in the tools and criteria used across studies^[7,27–31,33–38,41–44,47–49,51,56]^, with some defining visual improvement based solely on symptomatic changes. Supplemental Digital Content Table S1, available at: http://links.lww.com/JS9/G640 summarizes postoperative visual outcomes in TSM patients treated with EEA or TCA.

**Table 1:** Characteristics of the studies included for meta-analysis.

First author (year of publication)	Country	Total number of pts	Procedure	No. of patients in each group	Age, years	%Male	Mean follow-up time, months	Visual loss, *n* (%)	Headache, *n* (%)	Optic canal involvement, *n* (%)	Mean preoperative tumor volume, cm^3^	GTR, *n* (%)
Comparative study												
Carnevale (2024)	US	88	TCA	44	57.3 ± 14.1	26%	28 (2.1, 113.3)	25 (56)	25 (28)	NA	NA	37 (84)
			EEA	44				39 (89)		NA	NA	37 (84)
Marian-Magana (2024)	Mexico	29	TCA	16	56.6 ± 13.7	7%	NA	NA	NA	14 (87.5)	15.12 ± 9.18	NA
			EEA	13	43.5 ± 8.3	3%	NA	NA	NA	12 (92.3)	12.9 ± 7.93	NA
Mo (2024)	Japan	49	TCA	23	55 ± 14	26%	99 (1, 210)*	22 (96)	NA	NA	4 ± 5	16 (70)
			EEA	26	59 ± 14	31%	28 (0, 82)*	20 (77)	NA	NA	4 ± 3	16 (62)
Feng (2023)	China	84	TCA	39	53.1 ± 19.7	NA	49.1	33 (84.6)	15 (38.5)	21 (53.8)	10.6	34 (87.2)
			EEA	45		NA	42.6	38 (84.4)	19 (42.2)	27 (60)	11.3	41 (91.1)
Jiang (2023)	China	36	TCA	17	48 (32, 68)	11%	NA	NA	NA	NA	NA	16 (94.1)
			EEA	19			NA	NA	NA	NA	NA	18 (94.7)
Magill (2023)	US/Italy	947	TCA	629	54 (11, 90)	NA	26 (0, 265)	554 (88.0)	NA	NA	2.3 (0.3, 6.8)	448 (71.2)
			EEA	318		NA		246 (77.4)	NA	NA		217 (68.2)
Sakata (2023)	Japan	30	TCA	15	62.3 (35, 87)	30%	67.9 (8, 156)	15 (100)	NA	8 (53.3)	NA	10 (66.7)
			EEA	15				11 (73.3)	NA	13 (86.7)	NA	11 (73.3)
Alam (2022)	Bangladesh	34	TCA	29	NA	18%	66.1 (6, 120)	30 (88.2)	NA	NA	NA	24 (82.7)
			EEA	5	NA							3 (60)
Li (2022)	China	38	TCA	31	47.7*	40%	NA	27 (87.0)	NA	9 (29.0)	NA	27 (87.0)
			EEA	7			NA	5 (71.4)	NA	0 (0)	NA	6 (85.7)
Qian (2022)	China	112	TCA	78	51.0 ± 11.2	38%	20.5 (3, 36)	63 (80.8)	33 (42.3)	45 (57.7)	11.5 ± 4.6	67 (85.9)
			EEA	34				29 (85.3)	13 (38.2)	22 (64.7)	10.7 ± 5.2	31 (91.2)
Mallari (2021)	US	33	TCA	13	51 ± 9	15%	60 ± 42	8 (62)	NA	7 (54)	7.7 ± 8.5	5 (39)
			EEA	20	61 ± 12	5%	45 ± 44	17 (85)	NA	12 (60)	3.7 ± 3.5	16 (80)
Sankhla (2021)	India	62	TCA	38	41.9 (28, 73)	26%	NA	54 (87)	25 (40)	NA	NA	84.20%
			EEA	24			NA			NA	NA	87.50%
Kong (2019)	Korea	178	TCA	94	53.7 ± 11.0	25%	NA	77 (81.9)	2 (2.1)	51 (54.3)	NA	75 (79.8)
			EEA	84	54.2 ± 13.6		NA	80 (95.2)	1 (1.2)	60 (71.4)	NA	70 (83.3)
Kuga (2018)	Japan	20	TCA	13	56.1 ± 13.3	90%	32.4 (1.2, 81.6)	14 (70.0)	1 (5.0)	NA	3.7 (0.50, 6.87)	NA
			EEA	7								
Song (2017)	Korea	84	TCA	40	54.4 (24, 74)	NA	43.5	34 (85)	NA	32 (80)	5.8 ± 4.6	26 (68.4)
			EEA	44	52.7 (26, 76)	NA	27	44 (100)	NA	34 (77.3)	5.8 ± 3.4	37 (84.1)
Bander (2016)	US	32	TCA	15	55.0 ± 13.5	38%	37	8 (53)	NA	NA	5.04 ± 3.38	8 (53)
			EEA	17			25.06	15 (88)	NA	NA	5.58 ± 3.42	14 (82)
Fatemi (2008)	US	23	TCA	9	49 ± 7	NA	14 (3, 28)*	8 (89)	NA	NA	NA	NA
			EEA	14	51 ± 15	NA	27 (6, 65)*	11 (79)	NA	NA	NA	NA
de Divitiis (2008)	Italy	51	TCA	44	NA	NA	NA	NA	NA	NA	NA	39 (88.6)
			EEA	7	NA	NA	NA	NA	NA	NA	NA	6 (85.7)
Kitano (2007)	Japan	28	TCA	12	61.4 (47, 74)	17%	NA	NA	NA	NA	8.9 ± 9.4	NA
			EEA	16	53.8 (42, 76)	12%	NA	NA	NA	NA	7.5 ± 5.4	NA
One-arm study												
Bove (2024)	Italy	48	EEA	48	53.5 (26, 83)	27%	86.89 ± 54.06	41 (85.4)	NA	28 (58.3)	NA	40 (83.3)
Caklili (2023)	Turkey	60	EEA	60	50.3 ± 12.0	18%	45.42 ± 32.09	39 (65)	23 (38.3)	NA	NA	45 (75)
Henderson (2023)	US	23	EEA	23	NA	NA	NA	NA	NA	12 (52)	4.5	20/22 (91)
Zheng (2022)	China	19	EEA	19	56 (32, 37)	26%	NA	12 (63)	6 (31.6)	6 (31.6)	3.6 ± 2.5	17 (94.4)
Yu (2021)	China	40	EEA	40	58.3 (34, 82)	NA	NA	39 (97.5)	2 (5)	NA	NA	38 (95)
Elshazly (2018)	US	25	EEA	25	53.9 (26, 80)	16%	NA	20 (80)	4 (16)	17 (68)	5.29 (0.5, 28)	19 (76)
Zoli (2018)	Italy	35	EEA	35	58 ± 12.5	NA	NA	NA	NA	NA	NA	30 (85.7)
Hayashi (2017)	Japan	22	EEA	22	58.3 (32, 87)	32%	NA	18 (81.8)	4 (18.2)	7 (31.8)	NA	12 (54.5)
Khan (2014)	Canada	17	EEA	17	62 (37, 86)	NA	9.4 (3, 26)	11 (64.7)	NA	NA	NA	11 (64.7)
Koutourousiou (2014)	US	75	EEA	75	57.3 (36, 88)	19%	NA	61 (81.3)	13 (17.3)	NA	NA	57 (81.4)
Ottenhausen (2014)	US	20	EEA	20	56.5 (31, 81)	30%	NA	17 (85)	7 (35)	NA	11.98 (0.43, 28.93)	16 (80)
Ogawa (2012)	Japan	19	EEA	19	58.9 (43, 79)	26%	NA	NA	NA	NA	NA	NA
Wang (2010)	China	12	EEA	12	56.7	33%	25.2 (6, 60)	11 (92)	NA	NA	NA	11 (92)

Age, mean follow-up time, and mean preoperative tumor volume were presented as mean ± SD, median, median (range)*, or mean (range).

GTR, gross total resection; NA, not applicable; US, United States; SD, standard deviation; EEA, endoscopic endonasal approach; TSA, traditional transcranial approach.

### Risk of bias of the included studies

The quality of each individual study was assessed using the NOS for comparative studies and the Modified 18-item Delphi checklist for single-arm studies, with results presented in Supplemental Digital Content Table S1, available at: http://links.lww.com/JS9/G640. Overall, the NOS scores ranged from 6 to 9, indicating moderate to high quality for comparative studies, while the Modified 18-item Delphi scores ranged from 12 to 14, reflecting moderate quality for single-arm studies (Supplemental Digital Content Table S1, available at: http://links.lww.com/JS9/G640).

### Meta-analysis of visual outcomes

#### Visual outcomes after tumor resection via EEA

**Figure 2.**
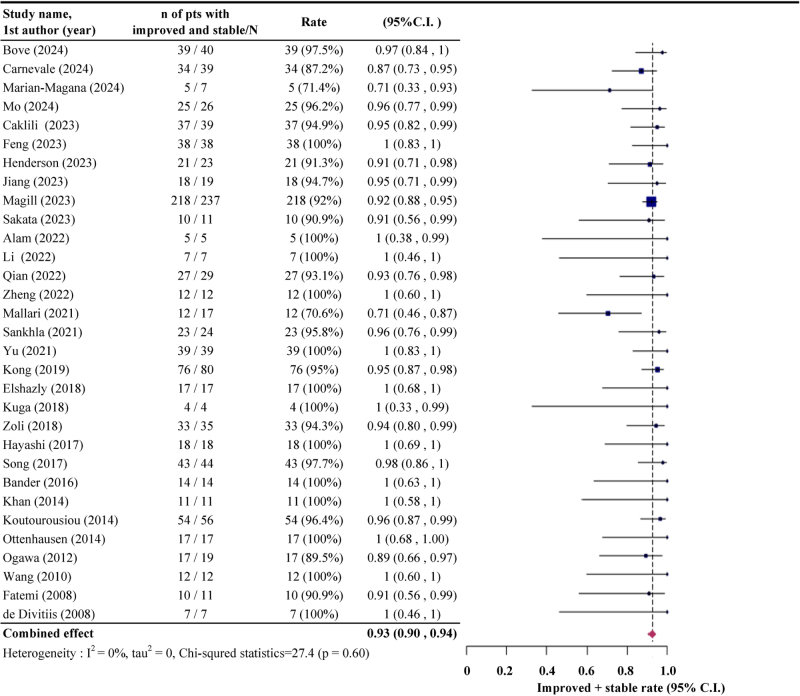
The pooled rate of improved or stable postoperative vision following endoscopic endonasal approach (EEA).

**Figure 3.**
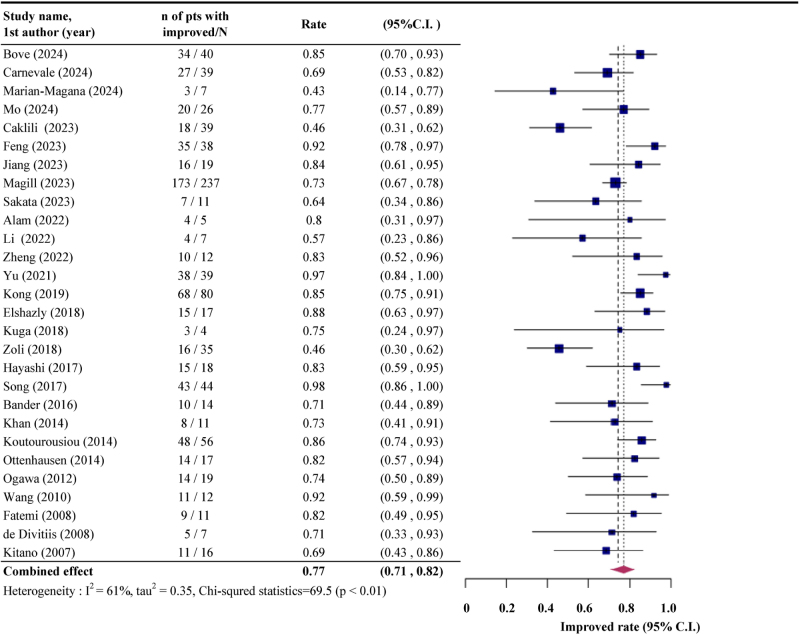
The pooled rate of improved postoperative vision following endoscopic endonasal approach (EEA).

Among the included studies, 31 provided data on the proportion of patients with improved or stable postoperative vision, while 28 specified visual improvement rates following EEA (Supplemental Digital Content Table S1, available at: http://links.lww.com/JS9/G640). The pooled rate of improved or stable postoperative vision was 0.93 (95% CI: 0.90, 0.94), calculated using both fixed-effects and random-effects models due to the absence of heterogeneity (*I*^2^ = 0%; *P* = 0.60; Fig. 2). For improved vision alone, the pooled rate was 0.77 (95% CI: 0.71, 0.82), derived using the random-effects model due to substantial heterogeneity (*I*^2^ = 61.1%; *P* < 0.01; Fig. 3).

### Comparing visual outcomes after resection via EEA and TCA

**Figure 4.**
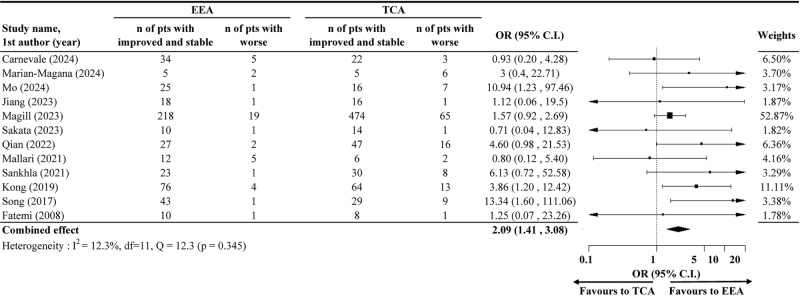
The odds of improved or stable postoperative vision after endoscopic endonasal approach (EEA) compared to after transcranial approach (TCA).

**Figure 5.**
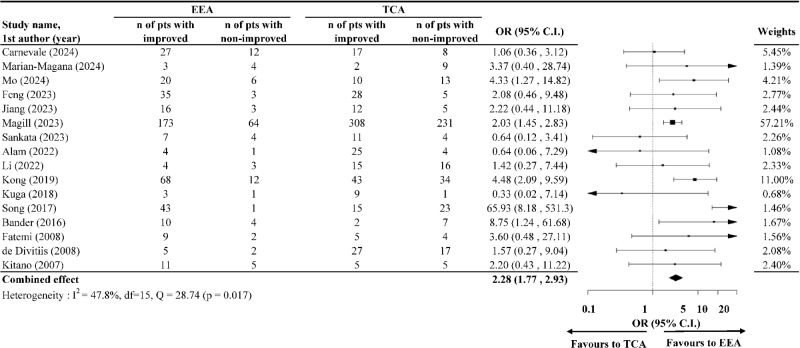
The odds of improved postoperative vision after endoscopic endonasal approach (EEA) compared to after transcranial approach (TCA).

Pooling data from 12 studies, the meta-analysis demonstrated that the odds of achieving visual improvement or stabilization were significantly higher with EEA than with TCA (OR = 2.09; 95% CI: 1.41, 3.08), with low heterogeneity (*I*^2^ = 12.3%, *P* = 0.345) detected (Fig. 4). For improved vision alone, the pooled OR was 2.28 (95% CI: 1.77, 2.93) based on results from 16 studies, with moderate heterogeneity (*I*^2^ = 47.8%, *P* = 0.017) detected (Fig. 5).

### Subgroup analysis, sensitivity analysis, and publication bias analysis for improved visual outcomes

Subgroup analyses were conducted to identify potential sources of heterogeneity among the 28 studies reporting visual improvement rates. Stratified by study region, 13 studies were conducted in Europe and the Americas, while 15 were from Asian countries. The pooled rate of improved vision after EEA was 0.71 (95% CI: 0.62, 0.79) for studies from Europe and the Americas, with moderate heterogeneity (*I*^2^ = 67.0%). Studies from Asian countries reported a higher pooled rate of 0.82 (95% CI: 0.77, 0.86), with relatively lower heterogeneity (*I*^2^ = 34.1%).

Stratification by publication year (before *vs* after 2015) revealed a pooled improvement rate of 0.79 (95% CI: 0.72, 0.85) for the eight studies published before 2015, with no heterogeneity (*I*^2^ = 0%). In comparison, the 20 studies published in 2015 or later had a pooled rate of 0.76 (95% CI: 0.68, 0.83), with substantial heterogeneity (*I*^2^ = 69.6%; Table 2).

**Table 2:** Subgroup analyses based on study country (Europe/Americas *vs* Asia) and study year (before *vs* after 2015).

	Number of studies	*Q* statistics	*I*^2^	Pooled rate with 95% CI
Total	28	69.5	61.1%	0.77 (0.71, 0.82)
Stratified by country				
Europe/Americas	13	36.4	67.0%	0.71 (0.62, 0.79)
Asia	15	21.3	34.1%	0.82 (0.77, 0.86) a
Stratified by publication year				
Before 2015	8	4.5	0%	0.79 (0.72, 0.85) a
After 2015	20	62.5	69.6%	0.76 (0.68, 0.83)

^a^ Fixed effect model.

Sensitivity analyses using the leave-one-out method were performed to assess the robustness of the meta-analysis. Sequential exclusion of each study did not result in significant deviations, with OR values ranging from 2.09 to 2.62 (Supplemental Digital Content Figure S1, available at: http://links.lww.com/JS9/G641). For instance, removing data from Song *et al* (2017) reduced heterogeneity to 0%, but the estimated OR remained at 2.19, consistent with the combined estimate and within the 95% CI. These results suggest that the meta-analysis was robust and not disproportionately influenced by any single study.

Publication bias analysis for improved postoperative vision (Supplemental Digital Content Figure S2, available at: http://links.lww.com/JS9/G641) indicated no evidence of bias, as confirmed by Egger’s test (*t* = 0.35, *P* = 0.733).

## Discussion

In this systematic review and meta-analysis, visual outcomes of TSM managed with EEA, with or without comparisons to the TCA, were evaluated. We examined visual evaluation metrics qualitatively and synthesized data to assess the proportion of patients experiencing improved or stabilized vision. Pre- and postoperative visual status was mainly assessed using visual acuity and visual field testing, often with guideline-endorsed tools. While most studies categorized visual results based on scores, some described postoperative change by symptoms. Notably, although individual studies may have applied their own standardized criteria, they did not report the operational definitions of “stable” or “improved” vision, and these definitions may have varied across studies. Despite these methodological variances, EEA demonstrated a high efficacy, with over 90% of patients achieving either improved or stabilized visual function. When focusing specifically on improvement alone, this rate approached 80%. Comparative analyses indicated that EEA was associated with a twofold higher likelihood of improving or stabilizing vision compared to TCA. Similar trends were observed in the odds of achieving visual improvement exclusively. These findings were further validated by subgroup analyses stratified by publication year and geographic region, as well as sensitivity analyses, which revealed limited publication bias and low-to-moderate heterogeneity across the included studies.

Prior meta-analyses have extensively examined surgical outcomes and complications associated with EEA. A recent comparative meta-analysis reported a pooled visual improvement rate of 86.6% for EEA, which was 3.5 times higher than that for TCA^[15]^. An earlier review by Yang *et al* (2019) demonstrated an 85.7% improvement rate for EEA based on six comparative studies, representing a fourfold advantage over TCA^[12]^. More recently, Lin *et al* (2023) highlighted the benefits of incorporating optic canal unroofing (OCU) with EEA, which resulted in a significantly higher improvement rate (88.4%) compared to OCU performed with TCA, particularly for smaller tumors^[13]^. Another meta-analysis identified EEA as the most effective surgical strategy for visual improvement in skull base meningiomas, with an 81.9% success rate for TSM, outperforming both the minimally invasive supraorbital approach assisted by endoscopy and the microscopic TCA^[14]^.

Of particular note, the findings by Khan *et al* (2021) closely paralleled our results, likely reflecting similarities in literature search methodologies^[14]^. Lin *et al* (2023) reported the highest improvement rates associated with EEA, potentially attributable to the inclusion of OCU in their analyses^[13]^. Importantly, none of the prior studies explicitly accounted for patients with stabilized postoperative vision or systematically reviewed the methodologies of visual assessments. A large-scale retrospective cohort study by Magill *et al* (2023) provided further corroboration, reporting that patients undergoing TSM resection via EEA exhibited superior or unchanged visual outcomes compared to those treated with TCA, which was particularly evident in individuals presenting with preoperative visual deficits^[7,8]^. However, this 40-site observational study also underscored the inconsistencies in documenting preoperative and postoperative visual deficits, with only 60% of patients undergoing formal visual field assessments despite receiving care at academic institutions^[7,8]^. This finding resonates with the variability observed in our systematic review.

Although this study does not delve into the mechanistic reasons underlying the superior visual outcomes associated with EEA compared to TCA, several plausible explanations have been proposed^[2,3,5,7]^. The EEA provides a direct, minimally invasive surgical route, obviating the need for cranial incisions and brain tissue retraction inherent to TCA. This approach affords an unobstructed operative field, facilitating meticulous evaluation of tumor extent, effective decompression of the optic nerves, and achieving GTR rates comparable to those seen with TCA. Given the proximity of TSMs to the optic chiasm, optic nerves, pituitary stalk, and diaphragma sellae, anatomical variation can critically influence EEA exposure and visual outcomes. Recent cadaveric work by Ay *et al* (2024) demonstrated substantial variability in the size, shape, and configuration of the foramen of the diaphragma sellae (FDS) – with irregular, circular, and oval morphologies – and identified normal, prefixed, and postfixed optic chiasm positions^[57]^. These variations can alter the working corridor and affect surgical maneuverability around neurovascular structures. A prefixed chiasm or irregularly shaped FDS may restrict operative visualization, whereas a postfixed chiasm may provide more favorable exposure of the tuberculum region. Such anatomical differences may also impact decisions regarding procedures such as OCU during decompression. Integrating this anatomical knowledge into preoperative planning may help optimize EEA trajectories, reduce surgical morbidity, and improve visual outcomes. These data highlight the importance of combining anatomical research with clinical outcome studies to refine minimally invasive skull base techniques.

We were unable to fully address the heterogeneity observed in the pooled rate of improved vision (*I*^2^ = 61.1%). Although subgroup analyses by geographic region and publication year were performed, several critical confounders – such as differences in patient selection, tumor characteristics, surgical approaches, follow-up duration, and methods of visual outcome assessment, including the use of techniques like OCU – were not accounted for. These variables were inconsistently reported across studies, could not be analyzed, and may represent unmeasured sources of heterogeneity. In contrast, some analyses in the present meta-analysis demonstrated no statistical heterogeneity (*I*^2^ = 0%), including the pooled rate of improved or stable postoperative vision after EEA and the subgroup of studies published before 2015. This indicates a high degree of consistency across studies in these analyses, despite differences in design, geographic setting, and institutional practices. The absence of heterogeneity strengthens the reliability of these pooled estimates and supports the generalizability of the findings across diverse clinical contexts. Advances in imaging modalities, surgical visualization, and closure techniques have further extended the scope of EEA to more complex cases, which may account for the consistent improvement rates across publication years observed in the current systematic review. Finally, our sensitivity analysis using the leave-one-out approach showed that exclusion of individual studies, including larger sample studies, did not substantially alter the pooled effect estimate, supporting the robustness of our findings. Notably, excluding Song *et al* – which reported an exceptionally high visual improvement rate in the EEA group – reduced heterogeneity to 0%, suggesting that it may have contributed to between-study variability. However, its characteristics did not appear markedly different from those of the other studies, so the observed influence may potentially result from differences in visual assessment.

Interestingly, studies conducted in Asian countries reported higher pooled visual improvement rates compared to those from Europe and the Americas – an unexpected finding given the presumed global standardization of endoscopic techniques. This regional disparity warrants cautious interpretation. Several unmeasured factors may have contributed to the observed difference. Variations in case volumes, surgical expertise, and institutional preferences – including the more frequent use of adjunct procedures such as OCU in certain Asian centers – may have influenced visual outcomes. It is also possible that differences in baseline tumor characteristics, such as tumor size, degree of optic nerve compression, or timing of intervention, affected the likelihood of postoperative improvement, although these parameters were not consistently reported across studies. Additionally, subtle differences in how visual improvement was defined or assessed – ranging from formal ophthalmologic testing to subjective clinical evaluations – may have introduced regional reporting biases. Furthermore, the observed regional differences in pooled improvement rates may reflect variation in surgical expertise, case selection, or institutional practice patterns, although these factors could not be directly assessed from the available data. The disparity underscores the need for standardized, prospective reporting of visual outcomes in future multicenter studies. Nevertheless, it should be acknowledged that these explanations remain speculative given the lack of granular, patient-level data.

These findings have meaningful clinical implications. Improvements in visual acuity or visual field – even if modest – can substantially enhance functional independence by facilitating reading, driving, fine-detail activities, and safe navigation in daily environments. Visual field gains are particularly important for reducing fall risk and improving mobility, while stabilization of vision alone may be clinically valuable for patients with progressive preoperative decline. Accordingly, the pooled visual outcomes in this meta-analysis carry direct relevance for postoperative quality of life and rehabilitation planning.

### Limitations

To our best knowledge, this study constitutes the most comprehensive systematic review and meta-analysis to date, providing robust pooled estimates on visual outcomes following TSM resection via EEA, either independently or in comparison to TCA. However, several limitations merit consideration. First, although the included studies were of moderate to high quality, their retrospective design may have introduced selection bias and reduced the reliability of the pooled estimates. The absence of RCTs represents a critical limitation, not only in the present review but also across the broader research landscape, precluding direct and unbiased comparisons. Prospective studies, ideally randomized or with rigorous standardized reporting frameworks, are needed to generate more definitive comparative evidence. Second, similar to prior meta-analyses, the heterogeneity in our analyses likely reflects differences in patient demographics, tumor characteristics, surgical techniques, follow-up duration, and outcome assessment methods, as well as variations in study design and operative protocols. For instance, differences in the extent of optic nerve invasion and the use of adjunct techniques such as OCU further complicate interpretation. Although the subgroup analyses provided partial insight into the observed heterogeneity, the insufficient and non-uniform reporting of key clinical variables across studies prevented the use of more advanced statistical techniques, such as meta-regression, to further explore potential sources of heterogeneity. Third, another major source of heterogeneity was the lack of standardized visual outcome definitions. The terms “improved” and “stable” vision were adopted directly from the included studies; however, these outcomes were often not clearly defined or standardized, which may limit the validity and reliability of our pooled estimates. Future investigations employing standardized outcome definitions are imperative to validate and refine these findings. Fourth, certain included studies reported findings that may reflect selection bias or atypical case inclusion. For example, no optic canal involvement was reported in the EEA group in one study, despite the focus on relatively large tumor volumes, raising the possibility of selection bias^[43]^. Nevertheless, its small sample size likely limits overall impact on the pooled results. Although Egger’s test and funnel plots showed no significant publication bias, excluding gray literature may still contribute to selective reporting^[13]^; however, we deliberately limited inclusion to peer-reviewed full-text articles to ensure methodological transparency, consistent reporting standards, and adequate data quality. Lastly, variations in patient populations, surgical approaches, and outcome reporting may have reflected institutional preferences or surgeon-specific practices. Several clinically relevant variables – such as tumor consistency, adhesion to surrounding structures, surgeon and institutional expertise, and intraoperative decision-making – were inconsistently or not reported, potentially influencing visual outcomes and contributing to residual heterogeneity despite efforts to synthesize comparable data across studies.

## Conclusion

This systematic review and meta-analysis underscore the effectiveness of the EEA in achieving favorable visual outcomes in patients with TSMs. With a high rate of vision preservation and improvement, EEA demonstrates its value as a minimally invasive surgical option. While comparative data suggest a trend toward improved visual outcomes with EEA compared to TCA, these findings should be interpreted with caution due to methodological limitations. We recommend that future RCTs or rigorously designed prospective studies use standardized ophthalmologic outcome measures and reporting to validate and refine these findings, deepen understanding of factors influencing visual recovery after TSM resection, and improve consistency, clinical interpretability, and bias mitigation through the incorporation of objective ophthalmologic testing (e.g., visual acuity and visual field assessments) and clear baseline comparisons.

## Data Availability

The datasets analyzed during the current study are available from the corresponding author on reasonable request.

## Research registration unique identifying number (UIN)

This systematic review and meta-analysis was registered in PROSPERO (registration number: CRD420251002845).
